# Preventable Mortality at Sea

**Published:** 1894-04-28

**Authors:** C. H. Leet

**Affiliations:** Seaforth, Liverpool


### Editor's Letter-Box.

[Our correspondents are reminded that proxility is a great bar to publication, and that brevity of style and conciseness of statement greatly facilitate early insertion.]

### PREVENTABLE MORTALITY AT SEA.

Sir,—I understand that in the new Shipping Bills provision will be made for checking the preventable waste of life at sea in the mercantile navy, which amounts to the lamentable average of 50 per cent., exclusive of "deaths from wrecks and casualties to ships." So I abandon my proposed public lectures on this important subject. To defray the expenses thereof I had advertised the pills prescribed in my private practice, but I have withdrawn every order for such publicity, being contrary to the bye-laws of the medical colleges, and the Royal College of Surgeons of England has accepted my apology and my promise to maintain the dignity of the College as one of their Fellows of long standing.

C. H. Leet, F.R.C.S.

Seaforth, Liverpool, 16th April, 1894.

⁂ We are glad to publish Mr. Leet's disclaimer, and hope that all his neighbours, professional and otherwise, will admit the excellence of his motives, though obliged to disapprove of some at least of the methods he now gives up. Mr. Leet committed an error of judgment, but an error of judgment is not a crime.—Ed. *T. H.*

